# Neoadjuvant apatinib combined with oxaliplatin and capecitabine in patients with locally advanced adenocarcinoma of stomach or gastroesophageal junction: a single-arm, open-label, phase 2 trial

**DOI:** 10.1186/s12916-022-02309-0

**Published:** 2022-04-06

**Authors:** Zhaoqing Tang, Yan Wang, Yiyi Yu, Yuehong Cui, Liang Liang, Chen Xu, Zhenbin Shen, Kuntang Shen, Xuefei Wang, Tianshu Liu, Yihong Sun

**Affiliations:** 1 Department of General Surgery, Zhongshan Hospital, Fudan University, 180 Fenglin Road, Shanghai, 200032 China; 2 Department of Medical Oncology, Zhongshan Hospital, Fudan University, 180 Fenglin Road, Shanghai, 200032 China; 3 Department of Radiology, Zhongshan Hospital, Fudan University, 180 Fenglin Road, Shanghai, 200032 China; 4 Department of Pathology, Zhongshan Hospital, Fudan University, 180 Fenglin Road, Shanghai, 200032 China

**Keywords:** Anti-angiogenics, Apatinib, Neoadjuvant therapy, Gastric cancer

## Abstract

**Background:**

Adding anti-angiogenics to neoadjuvant chemotherapy for localized gastric cancer is recognized as a promising strategy, but its clinical value remains to be defined.

**Methods:**

This single-center, single-arm, phase 2 trial included patients with locally advanced (cT3/4aN+M0) adenocarcinoma of the stomach or gastroesophageal junction (GEJ) who received three cycles of intravenous oxaliplatin (135 mg/m^2^ on day 1), oral capecitabine (1000 mg/m^2^ twice daily on days 1 to 14), and oral apatinib for 21 days (250 mg once daily in the first two cycles, and further increased to 500 mg daily in the third cycle based on whether any adverse event of grade 3 or worse occurred), and an additional cycle of oxaliplatin plus capecitabine, followed by gastrectomy with D2 lymphadenectomy. The primary endpoint was the proportion of patients who achieved an objective response according to RECIST version 1.1.

**Results:**

Between April 28, 2017, and October 23, 2019, 37 patients were screened and 35 participants were included. Of the 32 patients assessable for efficacy and safety, objective responses were achieved in 25 (78.1%; 95% confidence interval [CI], 60.0% to 90.7%) patients. Thirty-one (96.9%) patients received R0 resection, two (6.3%) patients achieved pathological complete response, and 11 (34.4%) patients achieved pathological response. At the data cutoff date (September 30, 2021), the median event-free survival was 42.6 (95% CI, 16.2 to not reached) months, and the median overall survival was not reached. The most common grade 3 or 4 treatment-emergent adverse events were hypertension (9/32, 28.1%), thrombocytopenia (7/32, 21.9%), and neutropenia (5/32, 15.6%). Seven (21.9%) patients developed surgical complications, and the most common one was intra-abdominal abscess (4/32, 12.5%).

**Conclusions:**

The concomitant use of apatinib, oxaliplatin, and capecitabine as neoadjuvant therapy showed promising efficacy and manageable safety profile in patients with locally advanced adenocarcinoma of the stomach or GEJ, and further phase 3 study is warranted.

**Trial registration:**

This study was registered with ClinicalTrial.gov (NCT03229096).

**Supplementary Information:**

The online version contains supplementary material available at 10.1186/s12916-022-02309-0.

## Background

Gastric cancer is one of the most common and fatal cancers in China [1]. Different to Japan and Korea, more than half of newly diagnosed gastric cancers in China are in advanced stage and have poor prognosis even after standard treatment [2]. The 5-year overall survival (OS) rate of gastric cancer is 93.6% in patients with early stage (pTNM IA) but drops dramatically to 17.9% in patients with more advanced stage (pTNM IIIC) [3]. The surgical resection, where primary tumor and regional lymph nodes were completely removed, is the fundamental management of standard treatment, but more extensive resection would not result in better prognosis for patients with locally advanced gastric cancer (LAGC) [4–6]. Thus, new treatment strategies are needed, and neoadjuvant chemotherapy is a choice. The JCOG0001 trial first used irinotecan and cisplatin preoperatively for patients with LAGC and extensive lymph node metastasis, which provided reasonable 3-year OS rate compared with historical data but caused three (5.4%) chemotherapy-related deaths [7]. The following JCOG0405 trial changed the neoadjuvant regimen to CS (S-1 and cisplatin), and the results showed better survival with acceptable safety profile [8]. In the Western countries, the FLOT4 trial used FLOT (fluorouracil plus leucovorin, oxaliplatin and docetaxel) as perioperative therapy for patients with LAGC, which brought higher pathological complete response (pCR) rate compared with the ECF (epirubicin, cisplatin and 5-fluorouracil)/ECX (epirubicin, cisplatin and capecitabine) group [9, 10]. However, not all the patients could gain benefit from neoadjuvant chemotherapy. In the FLOT4 trial, patients with diffuse type gastric cancer were less responsive to FLOT compared to those with non-diffuse type tumor. Moreover, the results of the JCOG0501 trial failed to show survival benefit of neoadjuvant CS for patients with Borrmann type 4 or large (≥ 8 cm) type 3 gastric cancer compared with upfront surgery [11]. New drugs or treatment regimens are still in need of development to achieve better response.

Angiogenesis is a hallmark in cancer and is responsible for invasive tumor growth and metastasis [12, 13]. Anti-angiogenic therapy, including anti-vascular endothelial growth factor (anti-VEGF) antibodies and tyrosine kinase inhibitors (TKIs), has been proved effective in many types of cancers, including gastric cancer [14–16]. Increasing evidence has suggested that moving the anti-angiogenic agents from further-line treatment for chemo-refractory tumors to neoadjuvant therapy for resectable tumors is promising [17, 18]. The phase 2 results of RAMSES trial showed that the addition of vascular endothelial growth factor receptor-2 (VEGFR-2) inhibitor ramucirumab to neoadjuvant FLOT for patients with locally advanced esophagogastric adenocarcinoma significantly increased R0 resection rate with no improvement in pCR [19]. In the ST03 trial, the addition of bevacizumab, a monoclonal antibody against VEGF, to perioperative ECX did not improve OS in patients with potentially resectable esophagogastric adenocarcinoma [20]. Until now, no survival benefits were found when combining anti-angiogenic antibodies with neoadjuvant chemotherapy.

The efficacy of apatinib, a novel oral small-molecule TKI with highly-selective affinity to VEGFR-2, has already been proven in several malignant tumors [21–23]. In a phase 3 study, apatinib monotherapy significantly improved OS and progression-free survival (PFS) compared with placebo in patients with chemo-refractory advanced or metastatic adenocarcinoma of the stomach and gastroesophageal junction (GEJ) [16]. In another preliminary study that used S-1, paclitaxel and apatinib as conversion therapy for patients with initially unresectable gastric cancer, the results showed an overall response rate of 75% and a R0 resection rate of 64.2% [24]. Most of the neoadjuvant regimens that proved to be effective in LAGC were platinum-based (cisplatin or oxaliplatin), so we conducted a trial to explore the efficacy and safety of apatinib combined with oxaliplatin and capecitabine (XELOX) as neoadjuvant therapy for patients with locally advanced adenocarcinoma of the stomach or GEJ.

## Methods

### Study design and participants

This study was a single-arm, open-label, phase 2 trial conducted at the Department of General Surgery, Zhongshan Hospital, Fudan University. Patients with locally advanced adenocarcinoma of stomach or GEJ were recruited between April 2017 and October 2019.

Key inclusion criteria were as follows: (1) aged between 18 and 75 years; (2) histologically confirmed adenocarcinoma of stomach or GEJ (Siewert type II or III) staged as clinical T3/4aN+M0 according to AJCC 7th; (3) had regional lymph nodes metastasis, defined as at least one enlarged lymph node (≥ 1.5 cm in short-diameter) within the extent of D2 lymphadenectomy on contrast-enhanced computed tomography, which could make sure that patients had measurable lesions according to Response Evaluation Criteria in Solid Tumors (RECIST) version 1.1; (4) absence of peritoneal metastasis confirmed by staging laparoscopy; (5) Eastern Cooperative Oncology Group (ECOG) performance status of 0–2; and (6) adequate bone marrow function (white blood cell count of ≥ 3000 cells per μL, neutrophil count of ≥ 1500 cells per μL, and hemoglobin concentration of ≥ 8.0 g/dL), liver function (alanine aminotransferase [ALT] and aspartate aminotransferase [AST] lower than twice the upper limit of normal [ULN] and total bilirubin lower than ULN), and renal function (glomerular filtration rate ≥ 60 mL/min).

Key exclusion criteria were as follows: (1) known allergies to any of the excipients, (2) uncontrolled blood pressure, (3) evidence of active bleeding or bleeding tendency (international normalized ratio > 1.5), or (4) unstable angina or myocardial infarction within 6 months before recruitment.

The study was approved by the Ethics Committee of Zhongshan Hospital. Written informed consent was obtained from each patient. This study was registered with ClinicalTrial.gov (NCT03229096).

### Procedures

Eligible patients received a total of four 21-day cycles of neoadjuvant therapy, including three cycles of apatinib plus XELOX and one additional cycle of XELOX. The first three cycles consisted of intravenous oxaliplatin 130 mg/m^2^ on day 1, oral capecitabine 1000 mg/m^2^ twice daily on days 1 to 14, and oral apatinib on days 1 to 21. Apatinib 250 mg once daily was administered in the first two cycles. The dose of apatinib would be increased to 500 mg per day in the third cycle if no adverse events (AEs) of grade 3 or worse occurred; otherwise, apatinib would be kept at 250 mg per day or terminated. Apatinib dose reduction was also allowed during the third cycle, if any of the following situations occurred: (1) hand-foot syndrome or proteinuria of grade 3 or worse; (2) drug-induced uncontrollable hypertension; (3) intolerable fatigue, anorexia, or vomiting; and (4) any grade of bleeding. No apatinib was given in the fourth cycle.

Measurable disease was assessed before treatment. Tumor response according to RECIST version 1.1 was assessed by the investigator (Dr. Liang Liang) using contrast-enhanced computed tomography or magnetic resonance imaging after two and four cycles of neoadjuvant therapy, respectively. At the start of each cycle, patients were assessed according to vital sign, physical examination, complete blood count, serum biochemistry, tumor markers, and routine urine examination. At the completion of the first two cycles, the treatment response of neoadjuvant therapy was evaluated according to RECIST version 1.1. If the tumor regressed or was stable, another two cycles would be given; otherwise, the possibility of R0 resection will be evaluated. For progressive tumor, if R0 resection was possible, the following two cycles would be canceled and the surgery would be scheduled; otherwise, protocol treatment would be terminated. At the completion of all four cycles of neoadjuvant therapy, if R0 resection was considered difficult, the protocol treatment was terminated.

Surgery was scheduled within 3 to 6 weeks after completion of the last cycle of neoadjuvant therapy, including gastrectomy and D2 lymphadenectomy. D2 lymphadenectomy was determined by the location and extent of the primary tumor according to Japanese gastric cancer treatment guidelines (4th edition) [25]. The pathological response of the tumor was evaluated according to Japanese classification of gastric carcinoma (3rd English edition). The tumor regression grade (TRG) was graded by the percentage of remaining viable tumor cells (grade 0–3: 100%, < 2/3, < 1/3, and 0%) [26]. Adjuvant chemotherapy was not specified, but four cycles of XELOX [27] were recommended.

AEs were monitored throughout the treatment period every 3 weeks at least and graded according to the Common Terminology Criteria for Adverse Events (version 4.0). The severity of surgical complications was graded according to the Clavien-Dindo classification [28].

### Outcomes

The primary endpoint was objective response rate (ORR), defined as the proportion of patients achieving complete response or partial response according to RECIST version 1.1. Considering that the primary gastric tumor was unmeasurable in most participants, the pretreatment enlarged lymph nodes were designated as measurable lesion. The secondary endpoints were safety, pathological response rate (pRR), 3-year event-free survival (EFS) rate, and 3-year OS rate. Safety included the incidence of treatment-emergent AEs and surgical complications. Pathological response was defined as less than 10% tumor cells residual in the specimen, and pathological complete response (pCR) was defined as an absence of tumor cells neither in the primary site nor in the resected lymph nodes. EFS was defined as the interval from the start of neoadjuvant therapy to tumor progression or death of any cause. OS was defined as the time from the start of neoadjuvant therapy to death of any cause.

### Statistical analysis

Based on the preliminary data of XELOX as perioperative therapy for patients with LAGC [29], the ORR of XELOX was about 50%. We expected that the ORR for apatinib combined with XELOX in the neoadjuvant therapy setting would increase from 50 to 75%. With a one-sided α error of 5% and a power of 80%, 31 patients were required. Assuming a dropout rate of 10%, the total sample size was 35.

Full analysis set (FAS) included all patients who received at least one dose of study drug. Surgery set included all patients who underwent surgery. Efficacy was analyzed in the FAS and surgery set. AEs during neoadjuvant therapy were analyzed in the safety set, including all patients who received at least one dose of study drug and safety evaluation. Surgical complications were analyzed in the surgery set. The surgery set and safety set were considered as the primary analysis for the efficacy and safety endpoint, respectively.

All analyses were performed using Stata software, version 17 (StataCorp, LLC), and Prism, version 8.4.3 (GraphPad Software, LLC). Descriptive statistics of baseline and clinicopathological characteristics were performed. Continuous variables with normal distribution were presented as mean (standard deviation); non-normal variables were described as median (minimum to maximum range); categorical variables were expressed as number (percentage). The proportion of patients with different response was calculated with 95% confidence interval (CI) by Clopper-Pearson method. The median EFS, median OS, 3-years EFS rate, and 3-year OS rate with corresponding 95% CIs were assessed by Kaplan-Meier method. Log-rank test was used to compare the EFS/OS between patients with different clinical or pathological responses, between patients with different grades of AEs, and between patients with or without apatinib dose escalation. All statistical tests were performed two-sided with a *p* < 0.05 considered statistically significant.

## Results

**Fig. 1:**
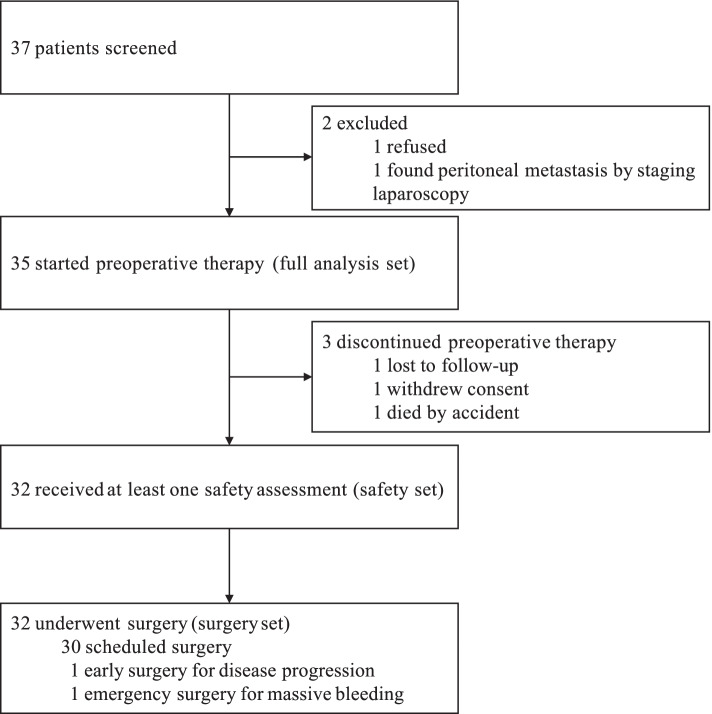
Flow chart

Between April 28, 2017, and October 23, 2019, 37 patients were screened, and 35 patients were enrolled and included in the FAS. One patient withdrew consent after the first cycle therapy, one died of car accident after first cycle therapy, and one lost follow-up during the first cycle therapy. All three of them did not receive any safety assessment, so they were excluded from the safety set. Thirty-two patients were eligible and assessable for the efficacy and safety of neoadjuvant therapy (Fig. 1). The median age was 62 years old (range, 35–73), and 77.1% of the patients were male. Most tumors were at cT4a (77.1%), cN2 (60.0%) stage, and located in stomach (68.6%). Detailed baseline characteristics were listed in Table 1.

**Table 1:** Baseline characteristics

Characteristics		FAS (*n* = 35)	Safety/surgery set (*n* = 32)
Age (years), median (range)		62 (35, 73)	60 (35, 73)
Gender, *n* (%)	Male	27 (77.1%)	26 (81.2%)
	Female	8 (22.9%)	6 (18.8%)
ECOG PS, *n* (%)	0–1	35 (100%)	32 (100%)
Tumor location, *n* (%)	Stomach	24 (68.6%)	21 (65.6%)
	GEJ	11 (31.4%)	11 (34.4%)
Histology, *n* (%)	Differentiated type	11 (31.4%)	10 (31.2%)
	Undifferentiated type	22 (62.9%)	21 (65.6%)
	NOS	2 (5.7%)	1 (3.1%)
Lauren subtype, *n* (%)	Intestinal	18 (51.4%)	17 (53.1%)
	Mixed	4 (11.4%)	4 (12.5%)
	Diffuse	8 (22.9%)	8 (25.0%)
	Unknown	5 (14.3%)	3 (9.4%)
Clinical tumor stage, *n* (%)	cT3	8 (22.9%)	8 (25.0%)
	cT4a	27 (77.1%)	24 (75.0%)
Clinical node stage, *n* (%)	cN1	11 (31.4%)	11 (34.4%)
	cN2	21 (60.0%)	20 (62.5%)
	cN3	3 (8.6%)	1 (3.1%)

*FAS* full analysis set, *ECOG PS* Eastern Cooperative Oncology Group performance status, *GEJ* gastroesophageal junction, *NOS* not otherwise specified

In the surgery set, regarding best objective response during neoadjuvant therapy, three (9.4%) patients showed complete response, 22 (68.7%) patients showed partial response, and seven (21.9%) patients showed stable disease. The detailed individual patient response and waterfall plot for tumor response are shown in Additional file 1: Fig. S1 and Additional file 2: Table S1, respectively. The ORR was 78.1% (95% CI, 60.0% to 90.7%). One patient was found serum CA199 re-elevated after the second cycle treatment and was considered tumor progression despite evaluation of stable disease according to RECIST version 1.1. Thus, the sequential cycles were discontinued, and the patient received gastrectomy.

All 32 patients proceeded to surgery, and R0 resection rate was 96.9% (31/32) since one patient only received explorative surgery owing to tumor invading the head of pancreas. One patient underwent emergency surgery because of acute life-threatening tumor bleeding. The pCR rate was 6.4% (2/31) in all resected patients and 6.3% (2/32) in the surgery set. The TRG according to Japanese Gastric Cancer Association were as follows: grade 1a (*n* = 12, 37.5%), grade 1b (*n* = 4, 12.5%), grade 2 (*n* = 13, 40.6%), and grade 3 (*n* = 2, 6.2%). The pathological response was observed in 11 patients (34.4%; 95% CI, 18.6% to 53.2%). Similar results were found in the FAS (Table 2).

**Table 2:** Responses to neoadjuvant therapy

	FAS (*n* = 35)	Surgery set (*n* = 32)
RECIST		
Complete response, *n* (%)	3 (8.6%)	3 (9.4%)
Partial response, *n* (%)	22 (62.9%)	22 (68.7%)
Stable disease, *n* (%)	7 (20.0%)	7 (21.9%)
Progressive disease, *n* (%)	0	0
Objective response rate, % (95% CI)	71.4% (53.7% to 85.4%)	78.1% (60.0% to 90.7%)
Disease control rate, % (95% CI)	91.4% (76.9% to 98.2%)	100% (89.1% to 100.0%) *
Tumor regression grade, *n* (%)		
Grade 0	0	0
Grade Ia	12 (34.3%)	12 (37.5%)
Grade Ib	4 (11.4%)	4 (12.5%)
Grade II	13 (37.1%)	13 (40.6%)
Grade III	2 (5.7%)	2 (6.2%)
Pathological response, *n* (% [95% CI])	11 (31.4% [16.8% to 49.3%])	11 (34.4% [18.6% to 53.2%])

*One-sided

*FAS* full analysis set, *RECIST* Response Evaluation Criteria in Solid Tumors, *CI* confidence interval

In 31 patients underwent gastrectomy, 28 (90.3%) patients started adjuvant chemotherapy; among them, 15 (48.4%) patients received 4 cycles of adjuvant XELOX, 12 (38.7%) had dose reductions or cycle interruptions due to AEs, one (3.2%) had tumor recurrence during adjuvant therapy, and three (9.7%) refused adjuvant therapy.

**Fig. 2:**
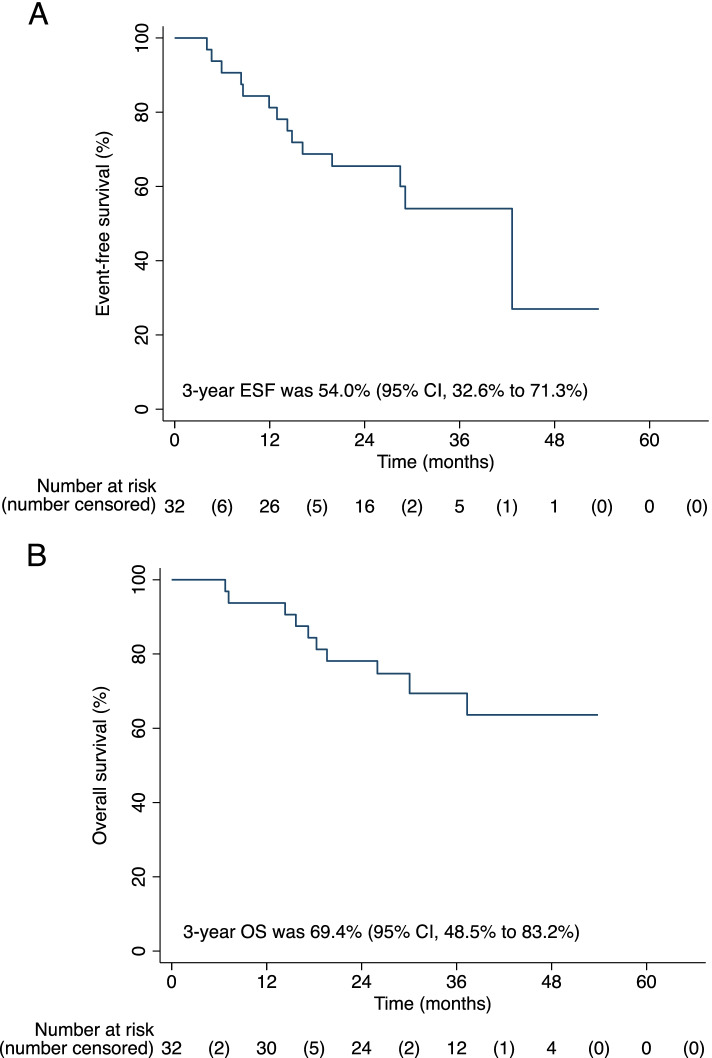
**A** Event-free survival (EFS) and **B** overall survival (OS) of patients in surgery set

At the data cutoff date on September 30, 2021, the median EFS follow-up was 31.7 months (range, 4.1–53.6), and median OS follow-up was 37.4 months (range, 6.8–53.9). Eleven patients had disease progression, among whom nine patients died. The 3-year EFS rate was 54.0% (95% CI, 32.6% to 71.3%) and 3-year OS rate was 69.4% (95% CI, 48.5% to 83.2%) (Fig. 2). Patients with better clinical responses or pathological responses appeared to have better survivals (Additional file 1: Fig. S2).

The most common treatment-emergent AEs were thrombocytopenia (50.0%), hypertension (43.8%), and neutropenia (37.5%). Eighteen patients had AEs of grade 3 or 4, among which the most common were the same as aforementioned hypertension (28.1%), thrombocytopenia (21.9%), and neutropenia (15.6%). The grade 3 or 4 AEs related to anti-angiogenic agents were hypertension (28.1%), proteinuria (3.1%), hand-foot syndrome (3.1%), and tumor bleeding (3.1%) (Table 3). All patients recovered without severe sequelae. Seven (21.9%) patients developed surgical complications, and the most common one was intra-abdominal abscess (12.5%). No reoperations or deaths occurred (Table 4).

**Table 3:** Neoadjuvant treatment-emergent adverse events (safety set, *n* = 32)

Adverse event	Any grade	Grades 1–2	Grade 3	Grade 4
Thrombocytopenia, *n* (%)	16 (50.0%)	9 (28.1%)	4 (12.5%)	3 (9.4%)
Hypertension, *n* (%)	14 (43.8%)	5 (15.6%)	9 (28.1%)	0
Neutropenia, *n* (%)	12 (37.5%)	7 (21.9%)	4 (12.5%)	1 (3.1%)
Leukopenia, *n* (%)	11 (34.4%)	11 (34.4%)	0	0
Anorexia, *n* (%)	10 (31.4%)	7 (21.9%)	3 (9.4%)	0
Proteinuria,* n* (%)	9 (28.1%)	8 (25.0%)	1 (3.1%)	0
Anemia, *n* (%)	8 (25.0%)	8 (25.0%)	0	0
Fatigue, *n* (%)	7 (21.9%)	4 (12.5%)	3 (9.4%)	0
Oral mucositis, *n* (%)	6 (18.8%)	6 (18.8%)	0	0
Vomiting, *n* (%)	5 (15.6%)	5 (15.6%)	0	0
Hand-foot syndrome, *n* (%)	2 (6.3%)	1(3.1%)	1 (3.1%)	0
Elevation of ALT/AST, *n* (%)	2 (6.3%)	2 (6.3%)	0	0
Tumor bleeding, *n* (%)	1 (3.1%)	0	0	1 (3.1%)

*ALT* alanine aminotransferase, *AST* aspartate aminotransferase. Any grade adverse events occurring in > 5% of patients and all grade 3 or 4 adverse events were listed. No grade 5 adverse events occurred

**Table 4:** Surgical complications (surgery set, *n* = 32)

Complications	Any grade	Grade I	Grade II	Grade III
Intra-abdominal abscess, *n* (%)	4 (12.5%)	0	3 (9.4%)	1 (3.1%)
Wound infection (seroma), *n* (%)	1 (3.1%)	1 (3.1%)	0	0
Acute cholecystitis, *n* (%)	1 (3.1%)	0	1 (3.1%)	0
Chylous ascites, *n* (%)	1 (3.1%)	0	1 (3.1%)	0

No reoperations or surgery-related deaths occurred

Seven (21.9%) patients discontinued apatinib, of whom one patient discontinued due to life-threatening tumor bleeding during the second cycle of neoadjuvant therapy and transferred to emergency surgery, one was assessed as disease progression after two cycles, one discontinued due to uncontrolled hypertension, two discontinued due to repeated grade 3 or 4 thrombocytopenia, and two discontinued due to prolonged elevation of ALT and/or AST. The dose of apatinib failed to increase to 500 mg per day in the third cycle therapy in ten patients (31.2%), and the most common reasons were hypertension, neutropenia, and intolerable fatigue or anorexia (Table 5).

**Table 5:** The reasons for apatinib dose change (safety set, *n* = 32)

Reasons	Dose escalation failure (*n* = 10)	Discontinuation (*n* = 7)
Hypertension, *n* (%)	5 (50.0%)	1 (14.3%)
Neutropenia, *n* (%)	4 (40.0%)	0
Thrombocytopenia, *n* (%)	2 (20.0%)	2 (28.6%)
Fatigue, *n* (%)	3 (30.0%)	0
Anorexia, *n* (%)	3 (30.0%)	0
Hand-foot syndrome, *n* (%)	1 (10.0%)	0
Elevation of ALT/AST, *n* (%)	0	2 (28.6%)
Emergency surgery, *n* (%)	0	1 (14.3%)
Disease progression, *n* (%)	0	1 (14.3%)

*ALT* alanine aminotransferase, *AST* aspartate aminotransferase

In a post hoc analysis of the association between apatinib dose and efficacy, or toxicity and efficacy, no statistically significant difference in survival outcomes were observed between patients who tolerated apatinib dose increasing from 250 mg to 500 mg per day or not (OS: hazard ratio [HR] = 0.692, 95% CI 0.194 to 2.46, *p* = 0.569; EFS: HR = 0.949, 95% CI 0.332 to 2.714, *p* = 0.922 ) (Additional file 1: Fig. S3), and there was no significant difference regarding survival outcomes between patients with or without apatinib-related AEs (OS: HR = 0.877, 95% CI 0.231 to 3.324, *p* = 0.846; EFS: HR = 0.786, 95% CI 0.262 to 2.360, *p* = 0.667) (Additional file 1: Fig. S4).

## Discussion

To our knowledge, this is the first study to evaluate the concomitant use of a VEGFR TKI and XELOX for patients with locally advanced adenocarcinoma of the stomach or GEJ in the neoadjuvant setting. Some similar studies also investigated neoadjuvant apatinib combined with chemotherapy (S-1 and oxaliplatin) but only reported short-term efficacy (R0 resection rate [30] or pRR [31]). Our study was first to report the survival outcomes in this setting. The results showed that 78.1% of patients achieved an objective response and 34.4% of patients achieved a pathological response. The 3-year EFS rate was 54.0% (95% CI, 32.6% to 71.3%) and 3-year OS rate was 69.4% (95% CI, 48.5% to 83.2%). Furthermore, this combination regimen was found to have an acceptable safety profile.

XELOX has already been proved to improve the survival prognosis in patients with stage II–III, resectable gastric cancer as adjuvant chemotherapy, and now XELOX is one of the standard adjuvant regimens after gastrectomy. For preoperative XELOX, an ORR of 49.0% in 48 patients with gastric cancer and para-aortic lymph nodes metastasis was reported in a study on conversion therapy [32]. In a phase 2 study of perioperative XELOX in 54 patients with LAGC, the ORR associated to preoperative XELOX was 50.0% [29]. Based on our hypothesis, we proved that the ORR of apatinib combined with XELOX was better than that of XELOX alone in the neoadjuvant setting.

Whether anti-angiogenic agents in the neoadjuvant setting can bring survival benefits to patients with gastric cancer was still controversial. For protein-based VEGF pathway inhibitors, until now, no evidence supported its application in the neoadjuvant setting. In the ST03 trial, the 3-year OS rate were 48.1% and 50.4% in the bevacizumab plus ECX group and the ECX group respectively (*p* = 0.36). Besides, the proportions of patients with objective response, pathological response and R0 resection associated to preoperative treatment were similar between the two groups [20]. In the RAMSES trial, preoperative ramucirumab plus FLOT lead to higher R0 resection rate than FLOT alone (97% vs. 83%, *p* = 0.0049), but pRR were similarly between the two groups (27% vs. 30%), while long-term survival outcome was to be waited [19]. However, both trials showed more severe treatment-related AEs and deaths. For VEGFR TKIs, two previously published studies reported the results of safety and efficacy when adding apatinib to preoperative S-1 and oxaliplatin (SOX) for LAGC. In the study by Zheng et al. which enrolled 29 patients with LAGC, the ORR was 79.3% and pRR was 37.9% [31]. In another study by Lin et al. which enrolled 48 patients with LAGC, the ORR was 75% and pRR was 25% [30]. In the present study, the ORR was 78.1% and pRR was 34.4%. These two surrogate endpoints of long-term outcomes were comparable in these three studies. Moreover, a prospective, multicenter, non-randomized, controlled trial of perioperative FLOT with or without apatinib for stage III gastric cancer in Chinese population is underway. The preliminary results demonstrated high R0 resection rate and manageable toxicity with neoadjuvant FLOT plus apatinib [33].

A previous study enrolled 54 patients with LAGC treated with perioperative XELOX alone [29]. The 3-year OS rate was 47.2% (95% CI, 33.4% to 59.8%) with XELOX in that study and higher with apatinib plus XELOX (69.4%, 95% CI 48.5% to 83.2%) in our study (Additional file 1: Fig. S5). Although cross-trial comparison was difficult and had less statistical power, we believe that adding apatinib to XELOX in neoadjuvant therapy might bring better survival to patients with LAGC.

Hypertension, proteinuria, and hand-foot syndrome are considered to be the most common AEs related to anti-angiogenic agents. In the present study, the incidence of hypertension, proteinuria, and hand-foot syndrome was 43.8%, 28.1%, and 6.3%, respectively, which were generally lower than those reported previously in patients with gastric cancer and ovarian cancer [22, 31]. This difference might be explained by the lower dose administered in our study (250–500 mg vs. 500 mg). The leading hematological AEs were thrombocytopenia and neutropenia, with incidence of 21.9% and 15.6% for grade 3 or 4 thrombocytopenia and neutropenia, respectively. The thrombocytopenia in our study was much higher than in other studies including XELOX alone as adjuvant or neoadjuvant chemotherapy, and apatinib alone as third-line chemotherapy [16, 27, 29]. Combination of apatinib and XELOX should be prescribed with caution for serious thrombocytopenia. Bleeding events were often observed in patients treated by anti-angiogenic agents. In the study by Li et al., the bleeding events were similar when patients with chemo-refractory gastric cancer treated by apatinib or placebo [16]. Life-threatening bleeding was common in patients with squamous cell carcinoma [34], but it was only reported by case in patients with gastric cancer [35]. One patient in our study suffered massive tumor bleeding during the second cycle of neoadjuvant therapy and received emergency gastrectomy after failure of non-surgical treatment. He was found peritoneal metastasis 3 months after surgery and died 3 months later. We would rather believe the bleeding was caused by tumor progression, though apatinib may contribute to the initiation of hemorrhage [36].

A previous clinical trial had suggested a daily dose of 850 mg apatinib in patients with chemo-refractory gastric cancer, but there was little evidence about the dose of apatinib when combined with chemotherapy at the time when we designed this study. Thus, our trial planned an initial dose of 250 mg/day in the first two cycles and a dose escalation to 500 mg/day in the third cycle if no AEs of grade 3 or 4 occurred. Only 78.1% of patients received three cycles of apatinib and 46.9% of patients tolerated dose escalation of apatinib. In a post hoc analysis, we found that patients who achieved dose escalation of apatinib had similar EFS and OS outcomes to those who failed dose escalation. Our results suggested that nearly half of the participants were not able to well tolerate apatinib of 500 mg/day when combined with chemotherapy and a higher dose of apatinib might not contribute to further improvement of survival outcomes. In a recent real-world study, low-dose apatinib of 250 mg/day was found to be effective and well-tolerated in patients with advanced gastric cancer [37]. Apatinib 250 or 375 mg/day was able to optimize tumor microenvironment and potentiate antitumor effect of PD-1/PD-L1 blockade [38]. Furthermore, apatinib 250 mg/day plus anti-PD-1 antibody in patients with non-squamous non-small cell lung cancer or patients with hepatocellular carcinoma showed acceptable toxicity and promising efficacy [39, 40]. Taking these findings into account, we suggest that the initial doses for apatinib should be 250 mg/day in further studies when combined with other regimens.

This study had some limitations. First, it was a single-arm trial without control group and thus selection bias could not be ruled out because of the non-randomized design. Moreover, the survival benefits from the addition of apatinib could not be concluded directly from this study. Second, the proportions of patients who tolerated the planned dose of apatinib was lower than expected, though it was partly due to the cautiously rules of dose escalation. We still provided valuable data to support the application of low-dose apatinib in the neoadjuvant setting for further studies.

## Conclusions

In conclusion, the combination of apatinib, oxaliplatin, and capecitabine had promising efficacy and manageable safety profile when used as neoadjuvant therapy in patients with locally advanced adenocarcinoma of the stomach or gastroesophageal junction, and further phase 3 randomized controlled trial is warranted.

## Supplementary Information

**Additional file 1: Figure S1.** Waterfall plot of tumor size change. **Figure S2.** Survival in different clinical/pathological responses. **Figure S3.** Survival in different doses of apatinib. **Figure S4.** Survival analysis for adverse events. **Figure S5.** Survival analysis for apatinib application.

**Additional file 2: Table S1.** Details about RECIST evaluation.

## Data Availability

Not applicable.

## Abbreviations

AEs: Adverse events
CI: Confidence interval
CS: S-1 and cisplatin
ECF: Epirubicin, cisplatin, and 5-fluorouracil
ECOG: Eastern Cooperative Oncology Group
ECX: Epirubicin, cisplatin, and capecitabine
EFS: Event-free survival
FAS: Full analysis set
FLOT: Fluorouracil plus leucovorin, oxaliplatin, and docetaxel
GEJ: Gastroesophageal junction
LAGC: Locally advanced gastric cancer
ORR: Objective response rate
OS: Overall survival
pCR: Pathological complete response
PFS: Progression-free survival
pRR: Pathological response rate
RECIST: Response Evaluation Criteria in Solid Tumors
SOX: Oxaliplatin and S-1
TKIs: Tyrosine kinase inhibitors
TRG: Tumor regression grade
VEGF: Vascular endothelial growth factor
VEGFR-2: Vascular endothelial growth factor receptor-2
XELOX: Oxaliplatin and capecitabine
