# Supplementary material for: Neoadjuvant apatinib combined with oxaliplatin and capecitabine in patients with locally advanced adenocarcinoma of stomach or gastroesophageal junction: a single-arm, open-label, phase 2 trial
Source: BMC Med. 2022 Apr 6;20:107. doi: 10.1186/s12916-022-02309-0 (PMC8985371; doi:10.1186/s12916-022-02309-0)
Supplement: Supplementary file 1 — Additional file 1: Figure S1. Waterfall plot of tumor size change. Figure S2. Survival in different clinical/pathological responses. Figure S3. Survival in different doses of apatinib. Figure S4. Survival analysis for adverse events. Figure S5. Survival analysis for apatinib application. [file 12916_2022_2309_MOESM1_ESM.pdf]

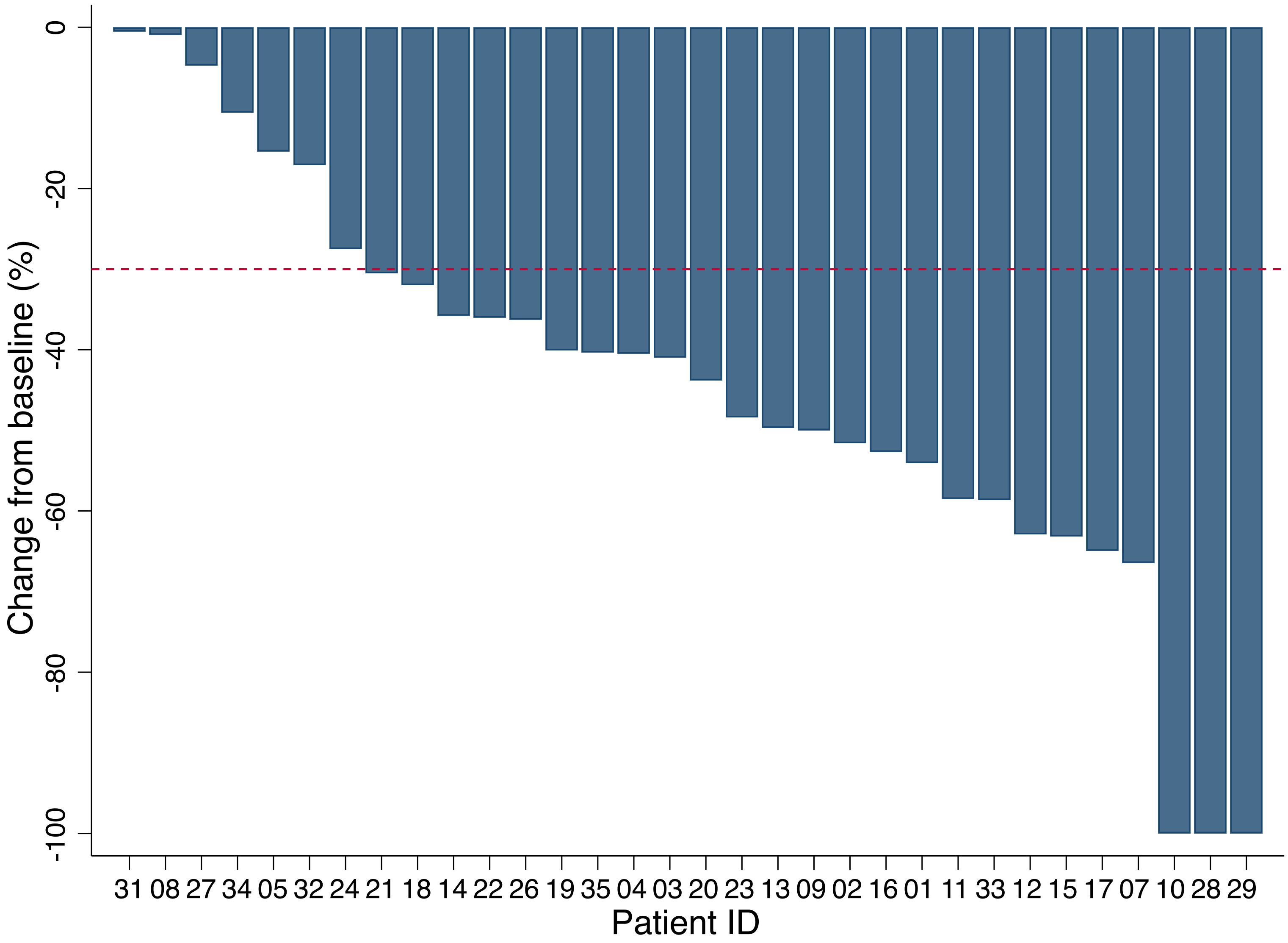

Figure S1. Waterfall plot of best change in target-lesion size from baseline of 32 eligible patients. Red dash line indicates the threshold of partial response (PR).

A

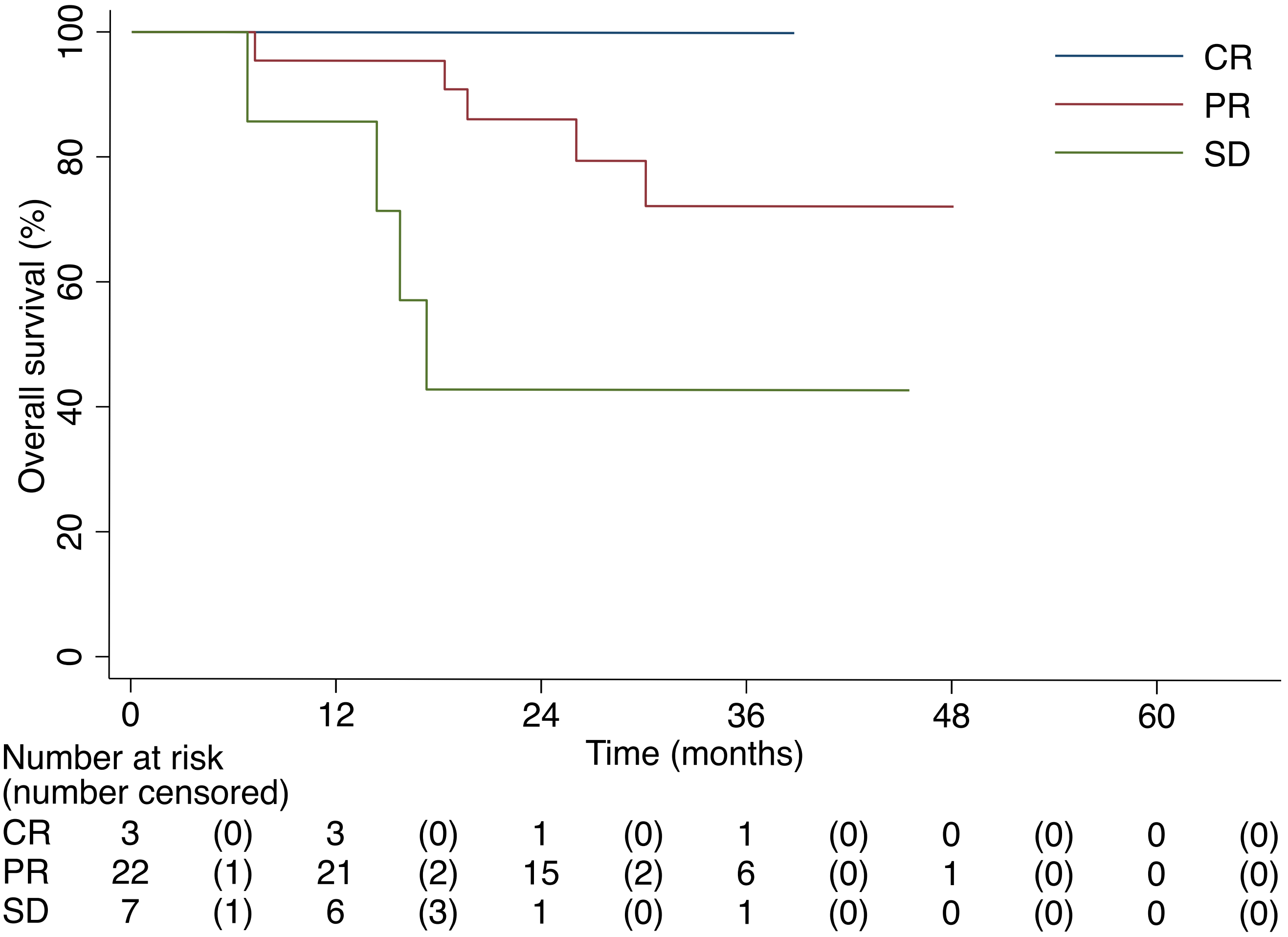

Number at risk (number censored)

	0		12		24		36		48		60	
CR	3	(0)	3	(0)	1	(0)	1	(0)	0	(0)	0	(0)
PR	22	(1)	21	(2)	15	(2)	6	(0)	1	(0)	0	(0)
SD	7	(1)	6	(3)	1	(0)	1	(0)	0	(0)	0	(0)

B

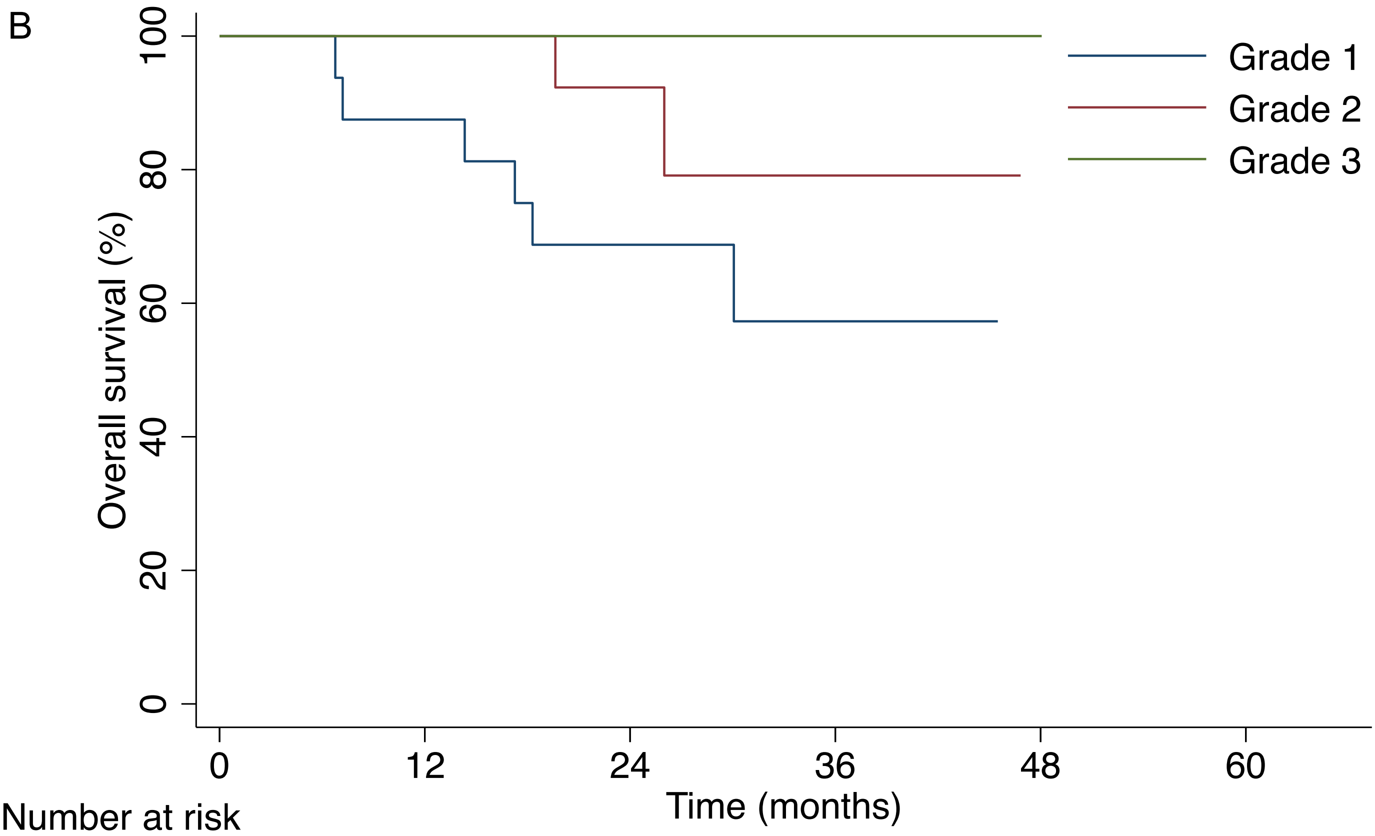

Number at risk (number censored)

	0		12		24		36		48		60	
Grade 1	16	(2)	14	(3)	7	(1)	3	(0)	0	(0)	0	(0)
Grade 2	13	(0)	13	(1)	8	(1)	3	(0)	0	(0)	0	(0)
Grade 3	2	(0)	2	(0)	2	(0)	2	(0)	1	(0)	0	(0)

Figure S2. Overall survival of patients in different clinical responses (A) and in different pathological responses (B). RECIST CR: complete response, PR: partial response, SD: stable disease. JGCA TRG grade 1: slight effect, grade 2: considerable effect, grade 3: complete response.

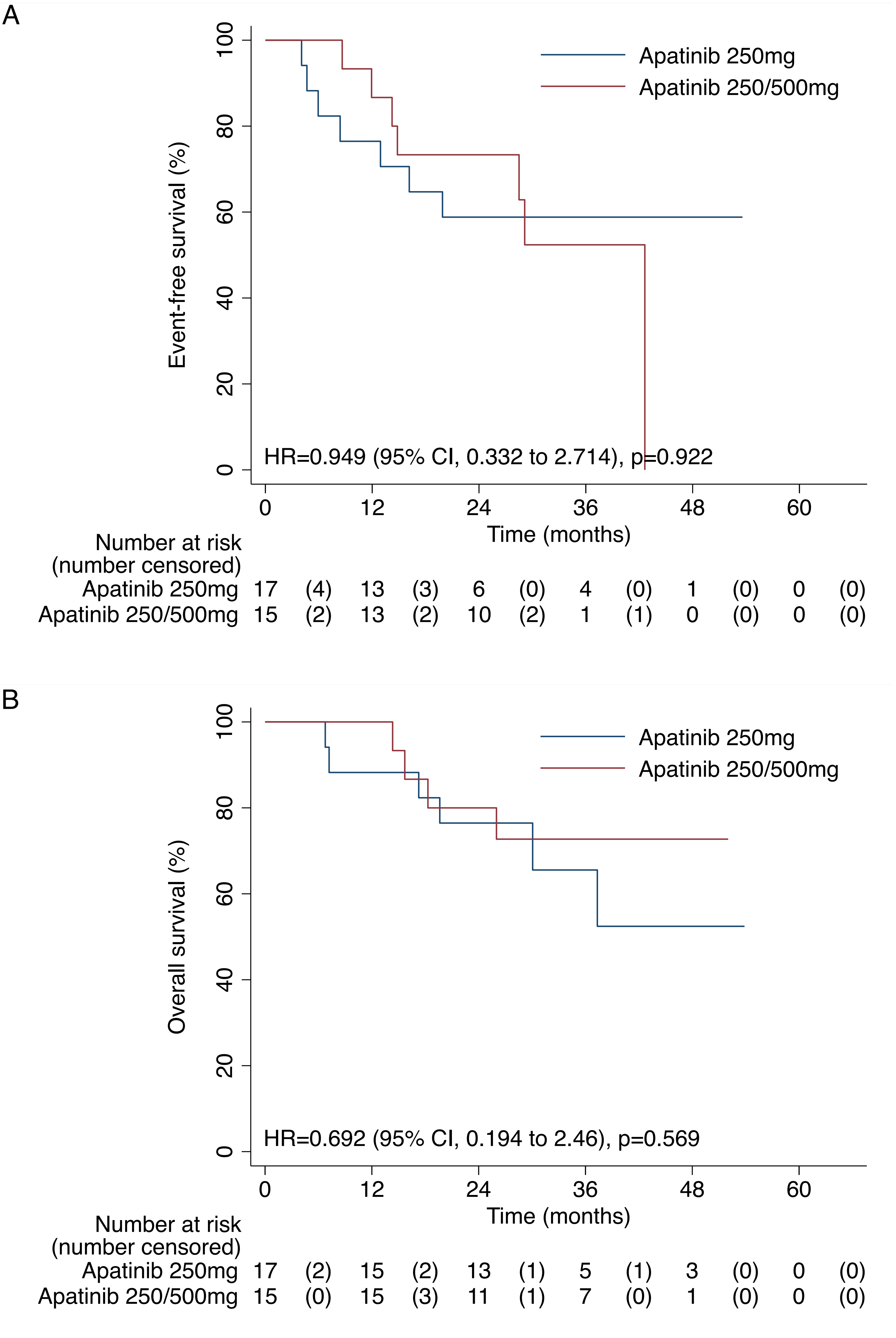

Figure S3. (A) Event-free survival (EFS) and (B) overall survival (OS) of patients received different doses of apatinib.

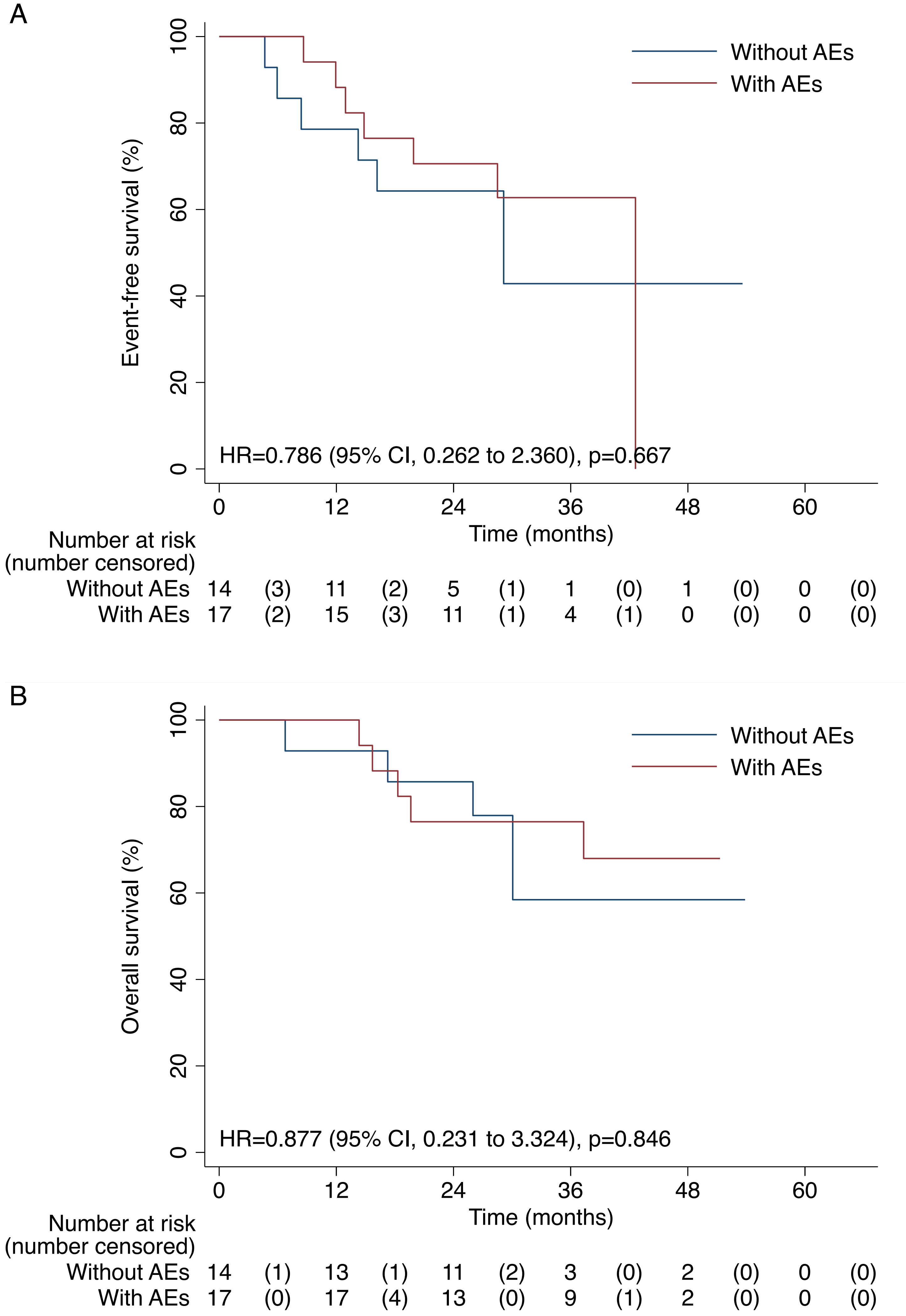

Figure S4. (A) Event-free survival (EFS) and (B) overall survival (OS) of patients with or without adverse events. AEs: adverse events.

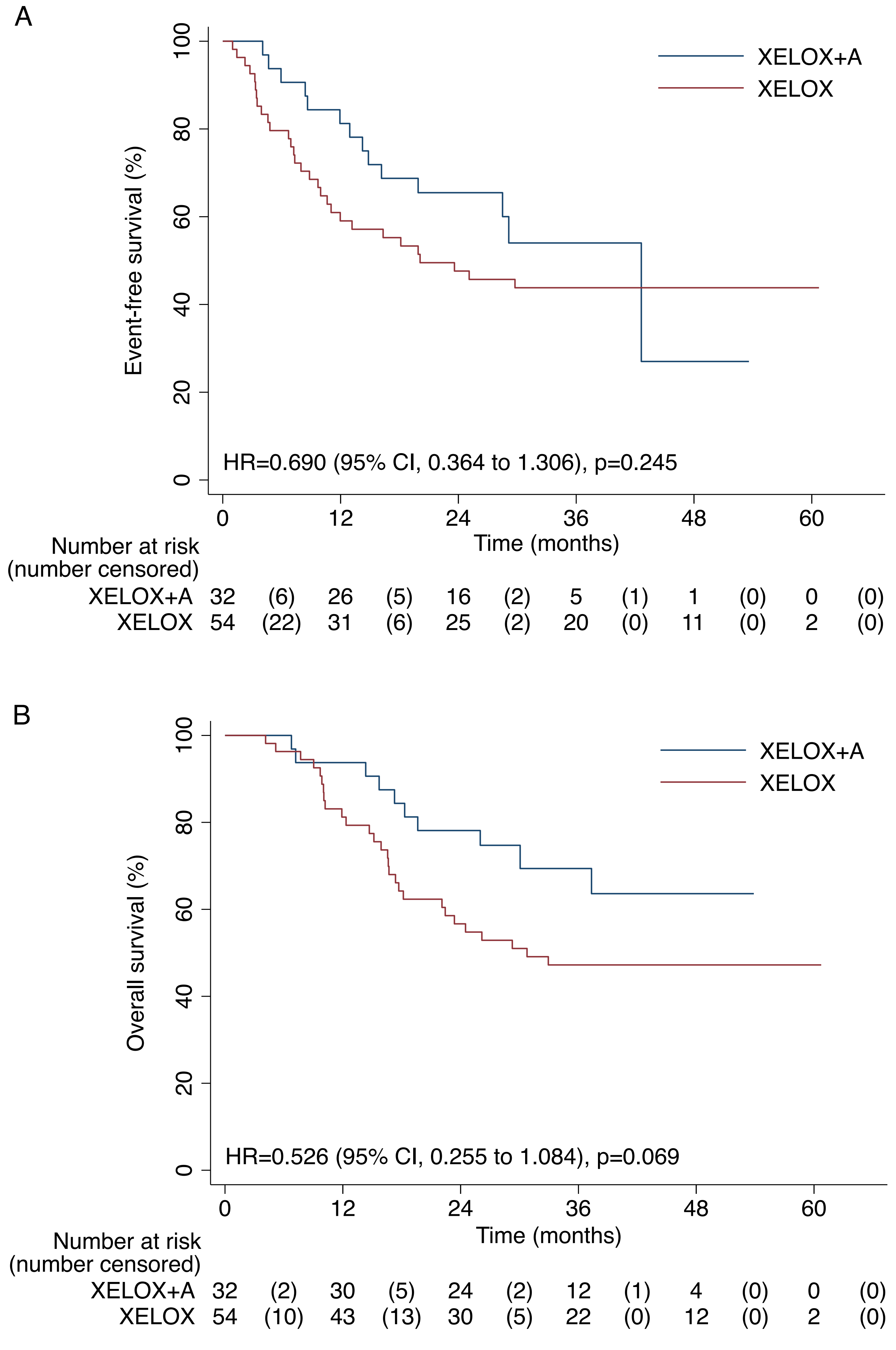

Figure S5. (A) Event-free survival (EFS) and (B) overall survival (OS) of patients received XELOX or XELOX plus apatinib. XELOX+A: XELOX plus apatinib; patients received XELOX alone from the NEO-CLASSIC study [29].
