# Supplementary material for: Neoadjuvant apatinib combined with oxaliplatin and capecitabine in patients with locally advanced adenocarcinoma of stomach or gastroesophageal junction: a single-arm, open-label, phase 2 trial
Source: BMC Med. 2022 Apr 6;20:107. doi: 10.1186/s12916-022-02309-0 (PMC8985371; doi:10.1186/s12916-022-02309-0)
Supplement: Supplementary file 2 — Additional file 2: Table S1. Details about RECIST evaluation. [file 12916_2022_2309_MOESM2_ESM.docx]

Table S1. Distribution of target lesions and associated RECIST grades among subjects.

| Case No. | Hospital ID | CT No. | Numbers of lymph nodes with size ≥ 3.0 cm^*^ | Numbers of lymph nodes with size ≥1.5cm, <3.0 cm^*^ | Percentage of Regression | RECIST 1.1^#^ |
| --- | --- | --- | --- | --- | --- | --- |
| AP01 | 1035588 | ZS17096653 | 0 | 1 (No. 9) | 54.1% | PR |
| AP02 | 1041507 | ZS17129893 | 1 (No. 7) | 1 (No. 4) | 51.6% | PR |
| AP03 | 1047132 | ZS17148933 | 0 | 1 (No. 11) | 41.0% | PR |
| AP04 | 1049102 | ZS17163557 | 2 (No. 6, 8a) | 0 | 40.5% | PR |
| AP05 | 1052013 | ZS17177337 | 0 | 1 (No. 9) | 15.4% | SD |
| AP06 | 1057239 | ZS17193751 | 0 | 1 (No. 6) | Excluded |  |
| AP07 | 1069070 | ZS17248731 | 1 (No. 3) | 5 (No. 4, 6, 8a, 9) | 66.5% | PR |
| AP08 | 1075254 | ZS17278743 | 1 (No. 6) | 0 | 1.0% | SD |
| AP09 | 1093504 | ZS18012419 | 0 | 1 (No. 3) | 50.0% | PR |
| AP10 | 1095855 | ZS18019960 | 0 | 1 (No. 1) | CR | CR |
| AP11 | 1108777 | ZS18071663 | 0 | 1 (No. 11) | 58.5% | PR |
| AP12 | 1112699 | ZS16257090 | 1 (No. 7) | 2 (No. 1, 3) | 62.9% | PR |
| AP13 | 1113437 | ZS18099619 | 1 (No. 7) | 0 | 49.7% | PR |
| AP14 | 1121380 | ZS18184603 | 0 | 1 (No. 11) | 35.8% | PR |
| AP15 | 1123139 | ZS18129027 | 0 | 3 (No. 1, 7) | 63.2% | PR |
| AP16 | 1130749 | ZS18175458 | 0 | 1 (No. 6) | 52.7% | PR |
| AP17 | 1136465 | ZS18202211 | 0 | 1 (No. 7) | 65.0% | PR |
| AP18 | 1142628 | ZS18228137 | 0 | 1 (No. 1) | 32.0% | PR |
| AP19 | 1145039 | ZS18238073 | 1 (No. 8a) | 2 (No. 3, 8a) | 40.1% | PR |
| AP20 | 1165625 | ZS18336024 | 0 | 2 (No. 6, 7) | 43.8% | PR |
| AP21 | 1197590 | ZS19106213 | 0 | 1 (No. 8a) | 30.5% | PR |
| AP22 | 1194796 | ZS19094166 | 1 (No. 11) | 1 (No. 7) | 36.0% | PR |
| AP23 | 1200817 | ZS19112603 | 0 | 1 (No. 8a) | 48.4% | PR |

Table S1. Distribution of target lesions and associated RECIST grades among subjects. (continued)

| Case No. | Hospital ID | CT No. | Numbers of lymph nodes with size ≥ 3.0 cm^*^ | Numbers of lymph nodes with size ≥1.5cm, <3.0 cm^*^ | Percentage of Regression | RECIST 1.1^#^ |
| --- | --- | --- | --- | --- | --- | --- |
| AP24 | 1201795 | ZS19123328 | 0 | 2 (No. 6, 8a) | 27.5% | SD |
| AP25 | 1204029 | ZS19131708 | 1 (No. 8a) | 0 | Excluded |  |
| AP26 | 1205145 | ZS19132159 | 0 | 2 (No.3) | 36.3% | PR |
| AP27 | 1205760 | ZS19140833 | 0 | 2 (No.3, 7) | 4.8% | SD |
| AP28 | 1210055 | ZS19168357 | 0 | 1 (No.8a) | CR | CR |
| AP29 | 1215382 | ZS19195062 | 0 | 1 (No.6) | CR | CR |
| AP30 | 1216355 | ZS19194845 | 1 (No.6) | 1 (No.6) | Excluded |  |
| AP31 | 1217977 | ZS19206633 | 0 | 1 (No.8a) | 0.5% | SD |
| AP32 | 1223115/1240865 | ZS19222633 | 0 | 1 (No.3) | 17.1% | SD |
| AP33 | 1223170 | ZS15291412 | 0 | 3 (No.1, 3) | 58.7% | PR |
| AP34 | 1234984 | ZS19282436 | 0 | 3 (No.1, 9, 11) | 10.6% | SD |
| AP35 | 1240941 | ZS19299231 | 0 | 2 (No.1, 4) | 40.4% | PR |

^*^The location of enlarged lymph node was listed in parentheses.

^#^RECIST CR: complete response, PR: partial response, SD: stable disease.
